# A Vector Fitting Approach for the Automated Estimation of Lumped Boundary Conditions of 1D Circulation Models

**DOI:** 10.1007/s13239-023-00669-z

**Published:** 2023-06-12

**Authors:** Elisa Fevola, Tommaso Bradde, Piero Triverio, Stefano Grivet-Talocia

**Affiliations:** 1 Department of Electronics and Telecommunications, Politecnico di Torino, Turin, Italy; 2 Department of Electrical & Computer Engineering, Institute of Biomedical Engineering, University of Toronto, Toronto, Canada

**Keywords:** Windkessel model, Vector fitting, Boundary conditions, Cardiovascular modeling, Circulation models

## Abstract

**Purpose:**

The choice of appropriate boundary conditions is a crucial step in the development of cardiovascular models for blood flow simulations. The three-element Windkessel model is usually employed as a lumped boundary condition, providing a reduced order representation of the peripheral circulation. However, the systematic estimation of the Windkessel parameters remains an open problem. Moreover, the Windkessel model is not always adequate to model blood flow dynamics, which often require more elaborate boundary conditions. In this study, we propose a method for the estimation of the parameters of high order boundary conditions, including the Windkessel model, from pressure and flow rate waveforms at the truncation point. Moreover, we investigate the effect of adopting higher order boundary conditions, corresponding to equivalent circuits with more than one storage element, on the accuracy of the model.

**Method:**

The proposed technique is based on Time-Domain Vector Fitting, a modeling algorithm that, given samples of the input and output of a system, such as pressure and flow waveforms, can derive a differential equation approximating their relation.

**Results:**

The capabilities of the proposed method are tested on a 1D circulation model consisting of the 55 largest human systemic arteries, to demonstrate its accuracy and its usefulness to estimate boundary conditions with order higher than the traditional Windkessel models. The proposed method is compared to other common estimation techniques, and its robustness in parameter estimation is verified in presence of noisy data and of physiological changes of aortic flow rate induced by mental stress.

**Conclusion:**

Results suggest that the proposed method is able to accurately estimate boundary conditions of arbitrary order. Higher order boundary conditions can improve the accuracy of cardiovascular simulations, and Time-Domain Vector Fitting can automatically estimate them.

## Introduction

Computational models of the cardiovascular system have become a valuable tool for the study and investigation of cardiovascular diseases [1]. Since a simulation of the entire cardiovascular system is computationally expensive, cardiovascular models usually include only a specific region of interest. The excluded regions are taken into account by choosing appropriate boundary conditions (BCs), which must provide a realistic representation of the haemodynamics in the rest of the circulatory system. Boundary conditions have been shown to largely affect flow rates, pressure distribution and important haemodynamic indicators, such as wall shear stress [2, 3]. For this reason, the selection of proper inlet and outlet boundary conditions that can realistically reproduce blood flow dynamics is particularly important. At the inlet, one typically imposes a flow rate waveform measured in vivo, while different solutions have been proposed as outlet boundary conditions [4]. Among these, the most commonly adopted (in order of increasing complexity) are:

- boundary conditions that simply prescribe a specific value for pressure or flow rate at the outlets [5];
- constant resistances, which result in a linear algebraic relation between pressure and flow rate [6–8];
- boundary conditions that impose a differential relation between pressure and flow rate, usually represented as equivalent lumped parameter networks. The latter can be classified according to their order, which corresponds to the number of storage elements (capacitors and inductors) present in the circuit, or equivalently to the order of the corresponding differential equation, see [9, Sect. 11.6]. A typical example is the three-element Windkessel model (3WK) [10], a circuit containing only one reactive component (the capacitor), and thus defined as a circuit of order one. Higher order Windkessel models, defined as circuits containing a total number of capacitors and inductors higher than one, have also been proposed [11].

The choice of the best model for outlet boundary conditions is generally the result of a trade-off between accuracy, model complexity, and number of parameters to estimate. The information available to estimate the boundary condition coefficients also plays a role. The most favourable scenario is when both flow rate and pressure information are available at the truncation location. In clinical practice this is not very common. While, with the advancements in MRI technology, flow rates are becoming increasingly available through phase contrast or 4D flow MRI, pressure information is harder to acquire in-vivo, typically requiring the use of catheters with pressure sensors. The most common scenario is when one or both quantities are not directly available, or are available at a different location (e.g. brachial pressure). In this case, the missing information is estimated using literature data, waveform generators [12, 13], or mathematical models for the systemic circulation [14]. In addition, several methods to recover pressure information from velocity-resolved MRI data have been recently proposed [15–17].

Presently, the most popular BC choice is the three-element Windkessel model, also known as RCR model (see "The Three-Element Windkessel Model" section). Even if the number of parameters in the Windkessel model is limited, obtaining an accurate estimate is not straightforward, due to the limited availability of in-vivo measurements. A simple, yet expensive approach consists in identifying reasonable ranges for each parameter, and then refining the choice by means of an iterative tuning procedure to obtain the desired pressure and flow rate [18]. If both pressure and flow data are available at the truncation location, more advanced and systematic approaches are generally used, where Windkessel models are fitted to available data. Note that, even if both pressure and flow rate data are known, it is not recommendable to impose them directly at the outlet sections since typical clinical measurements, due to uncertainty and acquisition errors, are typically incompatible with the computational model, and can result in numerical issues, especially in fluid–structure simulations. For example, flow rate measurements derived from phase contrast and 4D flow MRI images usually violate mass conservation to some extent [19].

Some common approaches for Windkessel parameter estimation are, for example, the simplex search method [10], or the least-square minimization [20]. Similarly, in [14], the terminal Windkessel resistances are estimated from mean pressure and outflow measurements at each terminal vessel, while terminal compliances are obtained by distributing the total peripheral compliance according to the cross-sectional areas of the outlets. The method proposed in [21], instead, selects parameters of the Windkessel models such that the net resistance and total compliance of the entire system are preserved. Other solutions resort to a non-iterative subspace model identification algorithm [22], or to other data-assimilation techniques, such as Kalman filtering [23, 24] and optimal control [25], but their applicability is limited by their high computational cost.

Overall, the existing solutions for the estimation of Windkessel parameters tend to be either empirical, or time consuming. Moreover, most of the available approaches are suitable only for the estimation of first order boundary conditions, such as the 3WK model, and are hard to generalize to higher order. Higher order BCs, in fact, have been proven to be more accurate and realistic than the three-element Windkessel model [10, 26], as they better capture the time evolution of pressure and flow rate, but the difficulty in estimating a larger number of parameters has limited their diffusion.

In this paper, we propose a novel approach for the automated estimation of boundary conditions of arbitrary order from pressure and flow rate waveforms co-located at the truncation point. Those waveforms may originate from literature data, waveform generators [12, 13], simplified models of the systemic circulation [21], in-vivo measurements where feasible, or a combination of these methods. The proposed method is based on and extends the Time-Domain Vector Fitting algorithm (TDVF), which approximates the behavior of a system by means of differential equations relating input and output [27, 28]. Unlike recently proposed approaches [29, 30], TDVF uses a model in the form of linear ordinary differential equations of small order, that can be easily generated and solved in real time with minimal computational efforts. Starting from pressure and flow rate waveforms at the truncation location, where the boundary condition must be imposed, TDVF can provide a boundary condition of arbitrary order relating pressure and flow rate very accurately. In the case of a model of order one, the proposed method provides an automated way to estimate the Windkessel parameters. For orders higher than one, instead, the model is represented as a differential relation between pressure and flow rate, which can be used as a boundary condition to Navier–Stokes equations, and is easy to implement in computational fluid dynamics (CFD) solvers. Also in the high-order case, model identification is fully automated.

It has been shown that TDVF can provide more accurate results with respect to other common modelling techniques [9], such as the autoregressive-exogenous (ARX) model adopted for example in [31], with improved scalability and robustness. In fact, Vector Fitting is ubiquitous in Electronic Design Automation tools. In this work, we assess the capability of the proposed TDVF method for cardiovascular applications on a 1D circulation model consisting of the 55 largest systemic arteries [32], by truncating some portions of the system and replacing them with boundary conditions of increasing order estimated with TDVF. Experimental results show that boundary conditions estimated with the proposed algorithm provide accurate pressure and flow rates at the truncation locations, making TDVF a promising candidate for parameter estimation in cardiovascular models. For Windkessel models, TDVF is compared to two other methods in the literature, one preserving the net resistance and total compliance of the original 55-artery system [21], and the other based on the Nelder-Mead simplex algorithm [33]. Overall, TDVF produces comparable, or better, results. The main advantage of the proposed approach is that it can easily estimate conditions of order higher than one, and results show that these can provide increased accuracy. Lastly, we verify that the proposed technique is able to accurately fit pressure and flow waveforms affected by noise down to 20 dB of signal-to-noise ratio, and that the estimated BCs remain valid in presence of physiological changes of the input waveforms (e.g., in case of mental stress [34, 35]).

## Methodology

**Fig. 1:**
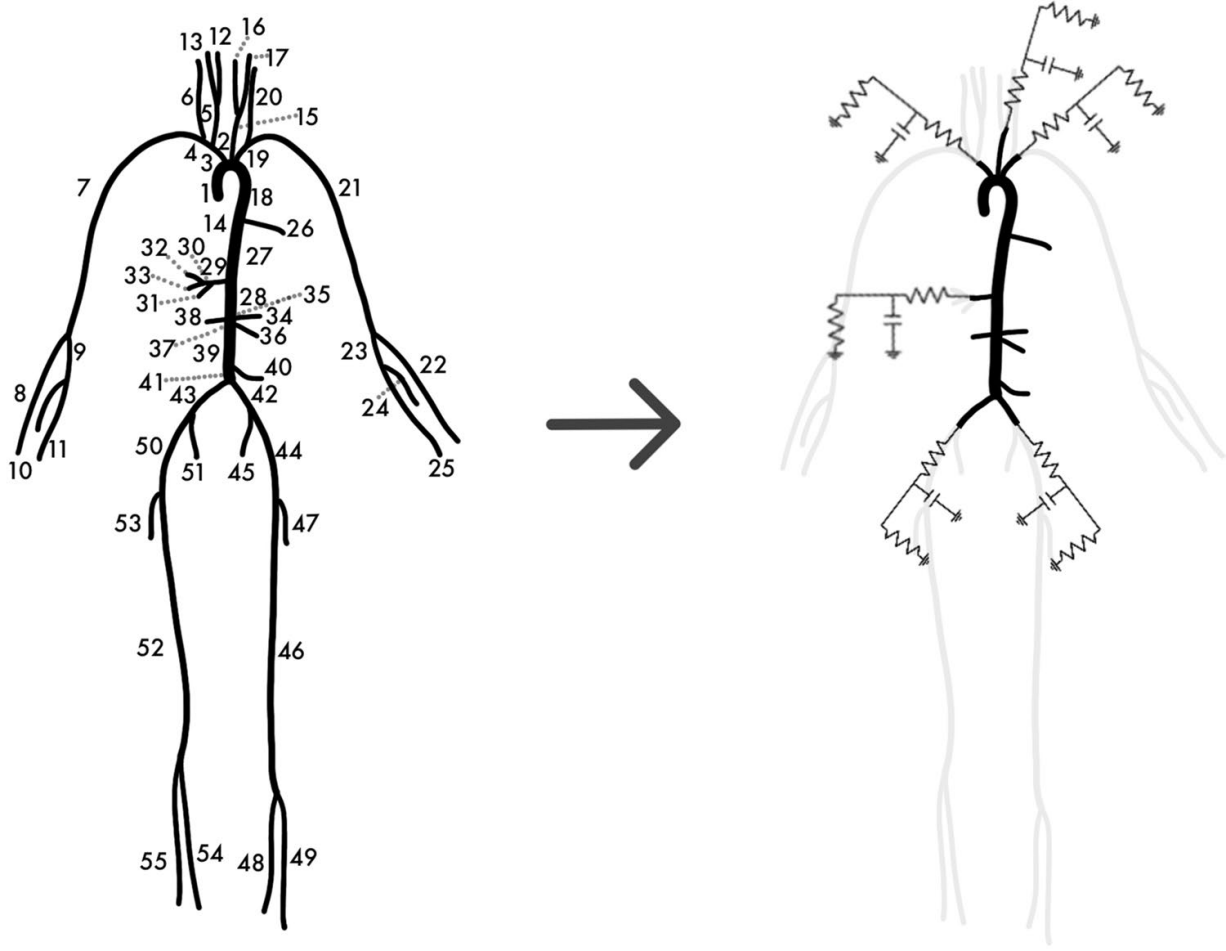
Left: schematic representation of the complete 55-artery network. Right: representation of the reduced model after boundary conditions estimation with Vector Fitting. The arterial segments are reduced from 55 to 21, and the truncated parts of the system (in grey) are substituted with the estimated boundary conditions. These could be Windkessel models, as depicted here, or models of higher order

In this section, the Time-Domain Vector Fitting algorithm will be introduced, together with the proposed formulation for boundary conditions estimation. The goal of this procedure is represented in Fig. 1, where we want to move from a model representing the systemic arterial system (Fig. 1, left), to a reduced version where part of the vasculature has been removed and substituted by properly estimated boundary conditions (Fig. 1, right). The latter could be Windkessel models, as displayed in Fig. 1, or general boundary conditions of higher order.

In the following subsections, first the three-element Windkessel model will be briefly reviewed ("The Three-Element Windkessel Model" section), and then a higher order generalization will be introduced, in a form suitable for the TDVF algorithm ("Generalization to High Order Boundary Conditions" section). The TDVF algorithm for the estimation of boundary conditions of arbitrary order will be presented in "Time-Domain Vector Fitting for Boundary Conditions Estimation" section, and the implementation of the obtained boundary conditions in 1D CFD solvers will be presented in "Implementation of High-Order Boundary Conditions" section.

### The Three-Element Windkessel Model

**Fig. 2:**
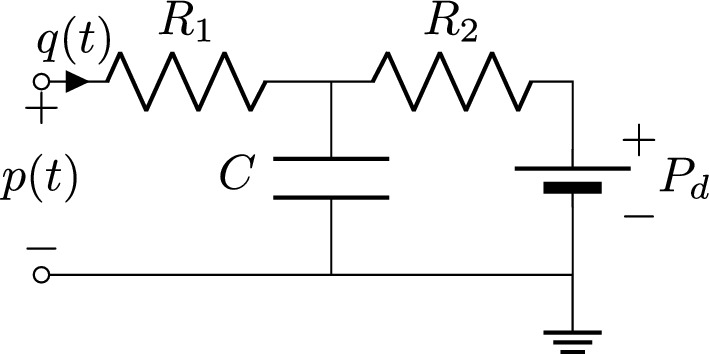
The three-element Windkessel model used as outlet boundary condition. The circuit includes the proximal resistance $R_1$, the distal resistance $R_2$, the capacitance *C* and the distal pressure $P_d$

The three-element Windkessel model was first introduced by Westerhof et al. [36]. Its circuit interpretation includes three elements, as displayed in Fig. 2: the capacitor *C* models the storage properties of arteries, the resistor $R_1$ represents the proximal resistance of the arterial network, while the resistor $R_2$ models the resistance of the distal circulation. Moreover, a distal pressure contribution $P_d$ is also included, in order to represent the pressure at which flow to the microcirculation ceases [32]. The Windkessel model relates pressure *p*(*t*) to flow rate *q*(*t*) by means of the differential equation

1 $$\begin{aligned} q(t)\left( 1+ \frac{R_1}{R_2}\right) + C R_1 \frac{d q}{d t} = \frac{p(t) - P_d}{R_2} + C\frac{d p}{d t}, \end{aligned}$$

whose derivation from the equivalent circuit of Fig. 2 is straightforward by exploiting the equivalence between fluid dynamics quantities (pressure, flow rate) and electrical quantities (potential, current). Estimating the Windkessel parameters consists in determining the optimal values for $R_1$, $R_2$, *C* and $P_d$ in (1) that best approximate the time domain evolution of the pressure and flow rate at the terminal point of the arterial network.

**Fig. 3:**
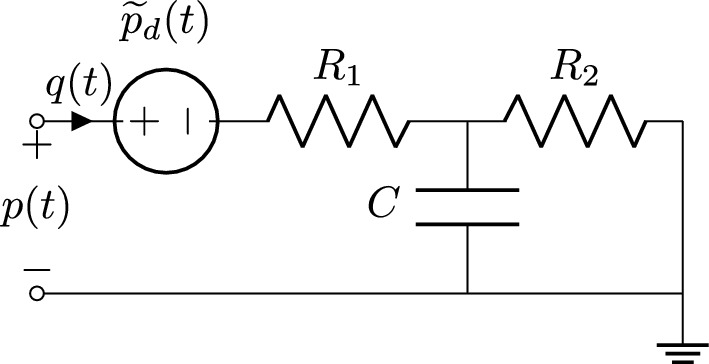
Equivalent Windkessel model, obtained by relocating the distal pressure contribution

**Fig. 4:**
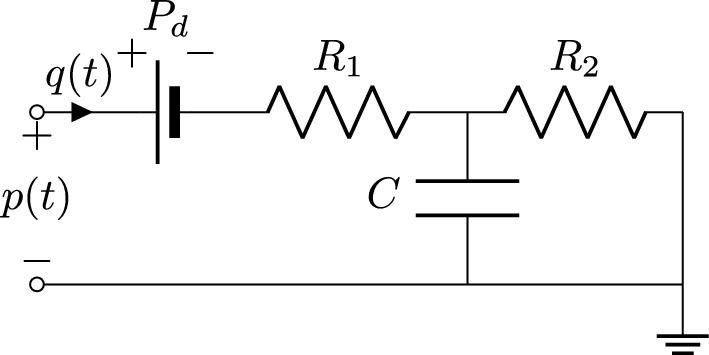
Approximate Windkessel model

#### Laplace-Domain Formulation

The following derivations and generalizations are best described in the Laplace domain [37]. The Laplace transform $\mathcal {L}$ is a standard mathematical tool that converts linear differential equations into algebraic equations, leading to a drastic simplification in both solution and interpretation of differential models.

Let us denote with *s* the Laplace variable (representing the time derivative operator *d*/*dt*), and define the Laplace transforms of *p*(*t*) and *q*(*t*) as *P*(*s*) and *Q*(*s*), respectively. Assuming vanishing initial conditions at $t=0$, the Laplace transform of (1) is

2 $$\begin{aligned} Q(s)\left( 1+ \frac{R_1}{R_2}\right) + s C R_1 Q(s) = \frac{P(s)}{R_2} -\frac{ P_d}{sR_2} + sCP(s), \end{aligned}$$

which is an algebraic relation between pressure and flow rate, parameterized by the constants $R_1$, $R_2$, *C*, and $P_d$. The distal pressure $P_d$ can be interpreted both as a free parameter, but also as an extra (constant) input, with specific reference to the circuit interpretation of Fig. 2 where it is represented as a voltage source.

Equation (2) can be rewritten as

3 $$\begin{aligned} P(s) = H(s)Q(s)+H_d(s)\frac{P_d}{s}, \end{aligned}$$

where *H*(*s*) and $H_d(s)$ are the two transfer functions

4 $$\begin{aligned} H(s)= R_1 + \frac{R_2}{sR_2C+1}, \quad H_d(s)= \frac{1}{sR_2C+1}. \end{aligned}$$

These are two first-order rational functions of the Laplace variable *s*, whose *order* is defined as the degree of the denominator. This is coherent with the differential equation (1), which includes only first-order derivatives. To enable the generalization proposed in this paper, we rewrite these transfer functions in the general pole-residue (partial fraction) form as

5 $$\begin{aligned} H(s)=c_0 + \frac{c_1}{s-a} \quad H_d(s)= \frac{b_1}{s-a}, \end{aligned}$$

where the pole *a*, the residues $c_1$, $b_1$, and the direct coupling constant $c_0$ can be uniquely related to the Windkessel parameters through

6 $$\begin{aligned} R_1 = c_0,\quad R_2=-\frac{c_1}{a}, \quad C=\frac{1}{c_1},\quad {\textrm{with}} \quad b_1=-a. \end{aligned}$$

### Generalization to High Order Boundary Conditions

In this section we show how (3) can be generalized to arbitrary order, in a way that will facilitate the estimation of its coefficients using the TDVF algorithm, presented in "Time-Domain Vector Fitting for Boundary Conditions Estimation" section. The proper derivation of the proposed high order boundary conditions, starting from the standard 3WK model, requires a number of steps, which are discussed in the three following sections. In particular, the first two steps eliminate the requirement of estimating two transfer functions, by modifying the structure of the boundary condition model to a single transfer function *H*(*s*). Finally, the third step generalizes *H*(*s*) to a high-order transfer function, which will be expressed for convenience in pole-residue form. We will see, in fact, that this model structure simplifies estimation of the parameters in "Time-Domain Vector Fitting for Boundary Conditions Estimation" section.

#### Relocation of the Distal Pressure Contribution

A well known result in circuit theory states that any linear and time invariant circuit with one port and internal sources can be transformed into an equivalent circuit, consisting of the series of an impedance and a voltage source. This result, known as Thevenin theorem [38], applies also to the present application case. When applied to the 3WK circuit of Fig. 2, we obtain the circuit of Fig. 3, where the equivalent source term is denoted as $\widetilde{p}_d(t)$. A full equivalence with the 3WK model is established in the Laplace domain by setting

7 $$\begin{aligned} \widetilde{P}_d(s) = H_d(s)\frac{P_d}{s} \end{aligned}$$

so that (3) can be restated as

8 $$\begin{aligned} P(s) = H(s)Q(s)+\widetilde{P}_d(s). \end{aligned}$$

With these definitions, the two circuits in Figs. 2 and 3 are indistinguishable in terms of the induced relationship between *P*(*s*) and *Q*(*s*).

Since $P_d$ is constant, applying the inverse Laplace transform to $\widetilde{P}_d(s)$ leads to

9 $$\begin{aligned} \widetilde{p}_d(t) = P_d \, \left( 1- e^{at} \right) \theta (t) \end{aligned}$$

where we used (5)–(6), and where $\theta (t)$ is the unit step (Heaviside) function. The signal $\widetilde{p}_d(t)$ converges exponentially to the asymptotic value $P_d$ with an initial transient, whose duration is related to the time constant $\tau = -1/a = R_2 C$.

#### Approximation of Early-Time Transient Behavior

The proposed generalization to higher order requires an approximation, which is motivated and discussed below. Although more advanced initialization schemes exist that result in shorter simulation times [39], time-domain cardiovascular simulations are often initialized to a vanishing initial state for all variables (pressure and flow rate in our case). However, the solution of practical and clinical interest is the periodic state operation that arises due to pulsating input excitation, which is usually applied in form of a predefined flow rate at the inlet. Such periodic state is reached after an initial transient, which is inevitably required by the numerical solvers, and which is generally disregarded when interpreting the results of the simulation.

Given the above observation, and noting that the equivalent source $\widetilde{p}_d(t)$ differs from its asymptotic value $P_d$ only during the initial transient, we replace $\widetilde{p}_d(t)$ with $P_d$ in the circuit of Fig. 3, obtaining the approximate Windkessel model depicted in Fig. 4. This operation corresponds to redefining $H_d(s)=1$ in (7), approximating then (8) as

10 $$\begin{aligned} P(s) \approx H(s)Q(s)+\frac{P_d}{s}. \end{aligned}$$

**Fig. 5:**
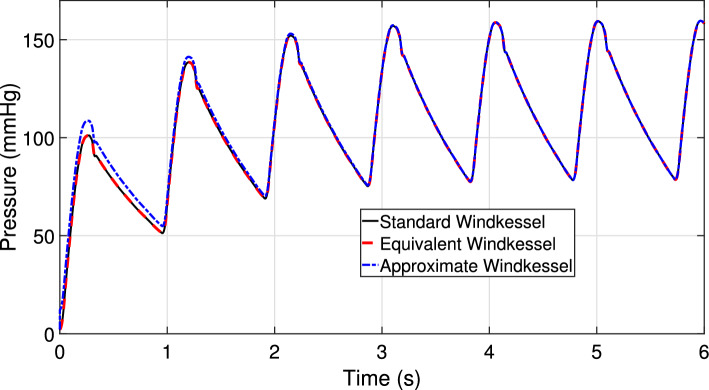
Pressure signals obtained by exciting the three boundary condition models: the standard Windkessel model of Fig. 2 (black line), the equivalent Windkessel model of Fig. 3 (red dashed line), and the approximate Windkessel model of Fig. 4 (blue line) with the same inlet flow excitation signal

A strict equivalence with the initial 3WK model of Fig. 2 no longer holds, but the only difference between the two formulations occurs at early times. When the initial transient is extinguished, the periodic states obtained with the two models are identical. This is confirmed by Fig. 5, where the pressure signals obtained by exciting the three models in Figs. 2, 3 and 4 with the same inlet flow excitation are depicted. The first two responses are identical in light of the full equivalence of the corresponding models. The response of the approximate model (blue line) asymptotically converges to the other two signals after the initial transient is extinguished. We conclude that, if only the periodic state operation is required, all discussed boundary condition models are equivalent.

#### Generalization to Arbitrary Order

Assuming the approximation discussed in "Approximation of Early-Time Transient Behavior" section, generalization to higher order boundary conditions becomes straightforward. We simply redefine the transfer function *H*(*s*) in (10) as a higher order rational function, expressed in pole-residue form as

11 $$\begin{aligned} H(s)=c_0 + \sum _{i=1}^n\frac{c_i}{s-a_i}. \end{aligned}$$

**Fig. 6:**
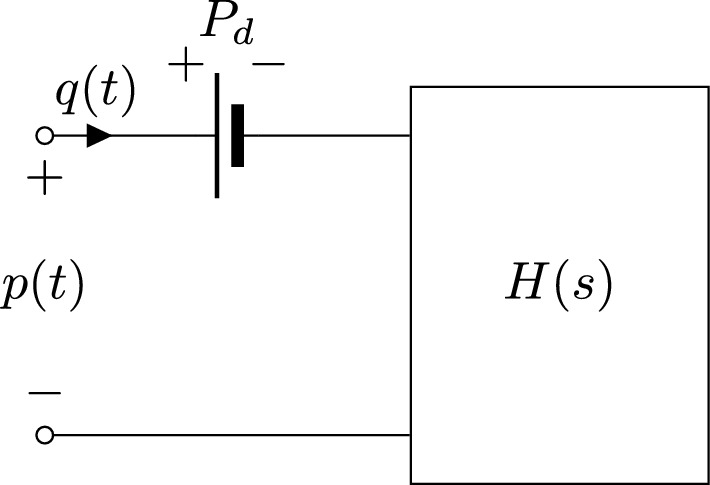
Proposed high-order boundary condition model

Based on (11), the representation (10) is easily converted to a set of coupled differential equations for direct inclusion as boundary conditions in 1D or 3D CFD solvers, see later "Implementation of High-Order Boundary Conditions" section. Therefore, the proposed high-order boundary condition model should be regarded as a black-box representation of the differential relation between outlet pressure and flow variables, as depicted in the model of Fig. 6. This model is characterized by richer dynamics and generally allows for more accurate numerical results compared to a first-order Windkessel model. These claims will be demonstrated by the numerical examples of "Results" section.

We close this section by providing an interpretation for the presence of *s* at the denominator associated to $P_d$ in (10). Since the Laplace transform of the unit step $\theta (t)$ is $\mathcal {L}\{\theta (t)\} = 1/s$, we see that the distal pressure term in (10) can be interpreted as a time-domain source $\widetilde{p}_d(t)=P_d\, \theta (t)$. Therefore, we see that the proposed high-order model assumes that the distal pressure contribution $P_d$ is applied instantaneously at $t=0$, rather than through an exponential transient (9). As discussed above, this difference is irrelevant when considering only the periodic state solution.

### Time-Domain Vector Fitting for Boundary Conditions Estimation

We now discuss how the parameters of the proposed high-order boundary conditions (10)–(11) can be automatically estimated from time series of pressure *p*(*t*) and flow rate *q*(*t*) at some vessel outlet. We assume that samples of these signals are available as

12 $$\begin{aligned} p(t_k),\;q(t_k), \quad k=0,\dots , K, \quad t_0=0, \end{aligned}$$

with a constant sampling rate $\Delta t = t_{k+1}-t_k$ and vanishing initial conditions $p(t_0)=q(t_0)=0$. Generalization to non-vanishing initial conditions will be provided in "Estimation in the Case of Non-zero Initial Conditions" section.

#### Model Parameterization

The proposed approach is based on the following model structure

13 $$\begin{aligned}&H(s)=\frac{N(s)}{D(s)}, \end{aligned}$$

14 $$\begin{aligned}&N(s)=c_0+\sum ^n_{i=1}\dfrac{c_i}{s-a_i} , \end{aligned}$$

15 $$\begin{aligned}&D(s)=d_0+\sum ^n_{i=1}\dfrac{d_i}{s-a_i} . \end{aligned}$$

The transfer function *H*(*s*) is expressed as a ratio of two rational functions sharing the same set of common poles $\{a_i\}$ with unknown residues $\{c_i\}$ and $\{d_i\}$. A simple algebraic simplification shows that these poles eventually cancel out: the poles $\{a_i\}$ are simply instrumental variables on which we construct the identification algorithm. The expressions (13)–(15) provide a parameterization of all proper rational functions with order *n*.

#### The TDVF Iteration

Let us start assuming that the poles $\{a_i\}$ in (14) and (15) are known, so that *H*(*s*) is parameterized only by the residues $\{c_i\}$, $\{d_i\}$ of numerator and denominator, respectively. These unknowns are computed by enforcing (10) as a fitting condition, based on the available pressure and flow samples. Using (13), we rewrite (10) as

16 $$\begin{aligned} D(s) P(s) \approx N(s) Q(s) + \frac{P_d}{s}D(s), \end{aligned}$$

which is obtained by multiplying both sides by the (unknown) denominator *D*(*s*). Plugging (14) and (15) into (16) leads to the relation

17 $$\begin{aligned}{} & {} \left( d_0+\sum ^n_{i=1}\dfrac{d_i}{s-a_i}\right) P(s) \approx \left( c_0+\sum ^n_{i=1}\dfrac{c_i}{s-a_i}\right) Q(s)\nonumber \\{} & {} \quad +P_d\left( \frac{d_0}{s}+\sum ^n_{i=1}\dfrac{d_i}{s\cdot (s-a_i)}\right) . \end{aligned}$$

The above can be expressed in time domain by applying the inverse Laplace transform to both sides, obtaining

18 $$\begin{aligned} d_0 \cdot p (t) + \sum ^n_{i=1} d_i \cdot {p}_i (t)&\approx c_0 \cdot q(t) + \sum ^n_{i=1} c_i \cdot {q}_i (t) \nonumber \\&\quad + P_d \,d_0 \cdot \theta (t) + \sum ^n_{i=1} P_d \, d_i \cdot {\theta }_i(t), \end{aligned}$$

where we used the shorthand notation

19 $$\begin{aligned} {z}_i(t) = \int _{0}^t e^{a_i(t-\tau )} z(\tau ) d \tau \end{aligned}$$

for any signal *z*(*t*). In order to render the approximation problem linear in the decision variables, we introduce the new set of dummy variables $b_i = P_d\, d_i$ and writing (18) for all discrete time samples $t=t_k$ leads to a homogeneous linear least squares problem in the unknowns $\{c_i\}$, $\{d_i\}$, $\{b_i\}$, which takes the compact form

20 $$\begin{aligned} A\,x\approx 0, \quad A=\begin{bmatrix} -\Phi \quad&\Gamma \quad&\Theta \end{bmatrix},\quad x=\begin{bmatrix} d \\ c \\ b \end{bmatrix}, \end{aligned}$$

where

21 $$\begin{aligned} \Phi&=\begin{bmatrix} p(t_0)&{}{p}_1(t_0)&{}\ldots &{}{p}_n(t_0)\\ \vdots &{}\vdots &{}\ddots &{}\vdots \\ p(t_K)&{}{p}_1(t_K)&{}\ldots &{}{p}_n(t_K) \end{bmatrix},&d=\begin{bmatrix} d_0 \\ d_1\\ \vdots \\ d_n \end{bmatrix}, \end{aligned}$$

22 $$\begin{aligned} \Gamma&=\begin{bmatrix} q(t_0)&{}{q}_1(t_0)&{}\ldots &{}{q}_n(t_0)\\ \vdots &{}\vdots &{}\ddots &{}\vdots \\ q(t_K)&{}{q}_1(t_K)&{}\ldots &{}{q}_n(t_K) \end{bmatrix} ,&c=\begin{bmatrix} c_0 \\ c_1\\ \vdots \\ c_n \end{bmatrix}, \end{aligned}$$

23 $$\begin{aligned} \Theta&=\begin{bmatrix} \theta (t_0)&{}{\theta }_1(t_0)&{}\ldots &{}{\theta }_n(t_0)\\ \vdots &{}\vdots &{}\ddots &{}\vdots \\ \theta (t_K)&{}{\theta }_1(t_K)&{}\ldots &{}{\theta }_n(t_K) \end{bmatrix},&b=\begin{bmatrix} b_0 \\ b_1\\ \vdots \\ b_n \end{bmatrix}. \end{aligned}$$

The solution of (20) is computed by enforcing $x\ne 0$ so that the trivial all-zero solution is avoided. This can be done e.g. by computing the Singular Value Decomposition (SVD) [40] of the matrix A,

24 $$\begin{aligned} A=U\Sigma V^T \end{aligned}$$

and choosing *x* as the last column of *V*, i.e., the right singular vector associated with the least singular value. Alternatively, a non-triviality constraint can be introduced in the problem, as in [41].

#### Pole Relocation

The above procedure determines the optimal set of coefficients $\{c_i\}$, $\{d_i\}$ given a prescribed set of numerator and denominator poles $\{a_i\}$, considered as known quantities. We now consider also these poles as unknowns to be determined. We will see below that the $\{a_i\}$ play the role of estimates for the poles of *H*(*s*), which are iteratively refined through a process denoted as *pole relocation* [9, 42].

An iteration with index $\nu$ is set up. At the first iteration $\nu =0$, the starting poles $\{a_i^{0}\}$ are initialized with a set of randomly distributed values throughout the expected frequency band of the model [42]. At any given iteration $\nu$, the set of current poles $\{a_i^{\nu }\}$ is used to construct and solve the least squares system (20). Let us denote as

25 $$\begin{aligned} D^{\nu }(s)= d_0^{\nu }+\sum ^n_{i=1}\dfrac{d_i^\nu }{s-a^\nu _i}\end{aligned}$$

the model denominator defined by the coefficients $\{d_i^\nu \}$ resulting from the least squares solution. Since the zeros of *D*(*s*) provide the poles of *H*(*s*), we define the poles for the next iteration as the zeros of $D^{\nu }(s)$

26 $$\begin{aligned} \{a^{\nu +1}\}=\{a_i: D^{\nu }(a_i)=0\}. \end{aligned}$$

These can be found algebraically by evaluating the poles of the inverse transfer function $[D^{\nu }(s)]^{-1}$. This is done by considering the state space realization of the denominator transfer function

27 $$\begin{aligned} D^{\nu }(s)\leftrightarrow (A^\nu , B^\nu , C^\nu , d_o^\nu ), \end{aligned}$$

where $A^\nu =\text {diag}\{a_1^\nu , \ldots , a_n^\nu \}$, $C^\nu =\left[ d_1^\nu , \ldots , d_n^\nu \right]$, and $B^\nu =\textbf{1} \in \mathbb {R}^n$ is a vector of ones. Algebraic inversion of the state space (27) shows that the poles of $[D^{\nu }(s)]^{-1}$ can be found by solving the eigenvalue problem

28 $$\begin{aligned} \{a^{\nu +1}\} = \lambda \{A^\nu - B^\nu (d_o^\nu )^{-1} C^\nu \}. \end{aligned}$$

For further details about the pole relocation steps see [9, 42].

In summary, the proposed algorithm involves solving (20) and redefining poles through (26) for $\nu =0,1,\dots$, until the set $\{a_i^{\nu }\}$ stabilizes, or until a maximum prescribed number of iteration $\nu _\textrm{max}$ is reached (in this work we set $\nu _{\textrm{max}}=100$). Under this convergence condition (see [9] for a detailed discussion), poles and zeros of $D^{\nu }(s)$ coincide so that $D^{\nu }(s)=d_0^{\nu }$, and the model (13) reduces to the numerator $N^{\nu }(s)$, characterized by poles $\{a_i^{\nu }\}$ and residues $\{c_i^{\nu }\}$. This algorithm can be regarded as an extension of the well-known TDVF scheme [27], suitably modified to account for the presence of the (unknown) distal pressure term, which produces the matrix block $\Theta$ and the additional dummy unknowns $\{b_i\}$ in (20).

#### Estimation of the Distal Pressure

Once *H*(*s*) is available from the above pole relocation iteration, the distal pressure $P_d$ can be determined in two alternative ways.

***From Least Squares Variables***

Recalling the definition of the dummy variables $b_i = P_d\, d_i$, and noting that both $\{b_i\}$ and $\{d_i\}$ are available from the least squares solution of (20), respectively collected in vectors *b* and *d*, we can determine $P_d$ as the least-square solution of

29 $$\begin{aligned} d\, P_d \approx b \quad \rightarrow \quad P_d = \frac{1}{\Vert d\Vert ^2} \,d^T \cdot b. \end{aligned}$$

***As Periodic State Bias***

From (10) we recall that

30 $$\begin{aligned} \frac{P_d}{s}\approx P(s)-H(s)Q(s), \end{aligned}$$

where the approximation becomes exact at periodic state, after the transient contribution of $P_d$ has extinguished. Suppose that the periodic state holds for $t\ge t_c$. We can thus find $P_d$ as the constant value that best fits the approximation

31 $$\begin{aligned} P_d \approx p(t) - p_m(t), \quad t\ge t_c, \end{aligned}$$

where

32 $$\begin{aligned} p_m(t)={\mathcal {L}^{-1}\{H(s)Q(s)\}} \end{aligned}$$

provides the output in absence of the distal pressure term. The best fit for $P_d$ is simply computed as the average

33 $$\begin{aligned} P_d=\frac{1}{K-c+1}\sum _{k=c}^K \left[ p(t_k)-p_m(t_k)\right] . \end{aligned}$$

#### Estimation in the Case of Non-zero Initial Conditions

When BC estimation is based on *in vivo* or generally real-time measurements, the assumption of vanishing initial conditions on the data samples is not realistic. Data recording starts at some time instant $t_0$, at which $q(t_0)\ne 0$ and $p(t_0)\ne 0$. In this case, the dynamic evolution of the pressure signal for $t\ge t_0$ includes not only the zero-state response analyzed in the foregoing sections, but also some contribution from the zero-input (natural) response [28]. The latter is due to the non-vanishing initial conditions on the internal system states of the underlying dynamical system, which are unknown. The following derivations show how to extend the proposed algorithm to handle also this situation.

The relation between pressure *P*(*s*) and flow rate *Q*(*s*) at the outlet can be generalized as

34 $$\begin{aligned} P(s) \approx H(s)Q(s)+G(s)+\frac{P_d}{s}, \end{aligned}$$

where *G*(*s*) represents the natural response contribution. The latter can be parameterized as

35 $$\begin{aligned} G(s)=\frac{B(s)}{s \cdot D(s)}, \quad B(s)=r_0+\sum ^n_{i=1}\dfrac{r_i}{s-a_i} \end{aligned}$$

based on the same starting poles $\{a_i\}$ and using the same denominator as in (13). This choice is motivated by the well-known fact that both input–output and natural response contributions of any linear time-invariant system share the same poles.

With these definitions, condition (34) is rewritten as

36 $$\begin{aligned}{} & {} \left( d_0+\sum ^n_{i=1}\dfrac{d_i}{s-a_i}\right) P(s) \approx \left( c_0+\sum ^n_{i=1}\dfrac{c_i}{s-a_i}\right) Q(s)\nonumber \\{} & {} \quad +P_d\left( \frac{d_0}{s}+\sum ^n_{i=1}\dfrac{d_i}{s\cdot (s-a_i)}\right) + \frac{r_0}{s}+\sum ^n_{i=1}\dfrac{r_i}{s\cdot (s-a_i)}, \end{aligned}$$

which replaces (17). The time domain equivalent is obtained by applying the inverse Laplace transform to both sides and collecting the common terms

37 $$\begin{aligned} d_0 \cdot p (t) +&\sum ^n_{i=1} d_i \cdot {p}_i (t) \approx c_0 \cdot q(t) + \sum ^n_{i=1} c_i \cdot {q}_i (t) + \nonumber \\&+ \underbrace{(P_d d_0+r_0)}_{b_0} \cdot \theta (t) + \sum ^n_{i=1}\underbrace{(P_d d_i+r_i)}_{b_i}\cdot {\theta }_i(t). \end{aligned}$$

When compared with (18), this expression differs only in the definition of the dummy variables $b_i$, that were nonetheless used only to estimate the distal pressure contribution after solving the least squares problem (20). For what concerns the estimation of the coefficients $\{c_i\}$ and $\{d_i\}$, the two problems (18) and (37) are identical. Therefore, the proposed estimation algorithm can be applied without any modification and independently on the conditions of the system when the recording of the training signals begins.

### Implementation of High-Order Boundary Conditions

Once the estimation process is completed, the obtained model can be used as a boundary condition in cardiovascular simulations. We already showed in "The Three-Element Windkessel Model" section how boundary conditions of order 1 can be represented as a three-element Windkessel model, and how it is possible to obtain Windkessel parameters from the general pole-residue form by means of (6).

For higher order BCs, different approaches can be adopted for their implementation into CFD solvers. One approach is to transform the final model expression (18) into an equivalent circuit by means of a synthesis process. Common techniques for equivalent circuit synthesis can be found in [9, 43]. An estimate of the required model order can be obtained through the algorithm proposed in [44]. An alternative approach consists in using directly the discretized differential equations obtained with TDVF as boundary conditions, without resorting to their equivalent circuit realization. Since the poles identified by TDVF could be either real or complex, the general transfer function (11) can be rewritten as

38 $$\begin{aligned} H(s) = c_0 + \sum _{i=1}^{n_r} \frac{c_{r_{i}}}{s-a_{r_{i}}} + \sum _{i=1}^{n_c}\bigg (\frac{c_{c_{i}}}{s-a_{c_{i}}} + \frac{c_{c_{i}}^*}{s-a_{c_{i}}^*}\bigg ), \end{aligned}$$

where the first sum includes the $n_r$ real poles, with $a_{r_{i}}, c_{r_{i}}\in \mathbb {R}$, while the second sum includes $n_c$ pairs of complex conjugate poles, with $a_{c_{i}}, c_{c_{i}} \in \mathbb {C}$, and where the superscript $^*$ denotes the complex conjugate. Multiplying (38) by flow rate *Q*(*s*) and using the inverse Laplace transform leads to a set of differential equations, which can be cast in the following state space form for real poles

39 $$\begin{aligned} {\left\{ \begin{array}{ll} \dot{x}_i(t)&{}=a_{r_{i}} x_i(t) + q(t)\\ p_r(t) &{}= \sum \nolimits _{i=1}^{n_r} c_{r_{i}} x_i(t) \end{array}\right. } \end{aligned}$$

and in the following form for complex pole pairs

40 $$\begin{aligned} {\left\{ \begin{array}{ll} \dot{x}'_i(t)&{}=\sigma _{c_{i}} x'_i(t) + \omega _{c_i} x''_{i}(t) + 2q(t)\\ \dot{x}''_{i}(t)&{}=-\omega _{c_i} x'_i(t) +\sigma _{c_{i}} x''_{i}(t) \\ p_c(t) &{}= \sum \nolimits _{i=1}^{n_c}(c_{c_{i}}' x'_i(t) + c_{c_{i}}'' x''_{i}(t)) \end{array}\right. } \end{aligned}$$

where $c_{c_{i}} = c_{c_{i}}' + j c_{c_{i}}''$ and $a_{c_{i}} = \sigma _{c_{i}} + j\omega _{c_{i}}$. Systems (39) and (40) are composed of linear differential equations, so in principle they can discretized with most algorithms suited for differential equations, including those typically used for 3D Navier–Stokes equations in CFD solvers. For a review of the methods and considerations pertinent to the coupling of 0D models to the 3D Navier–Stokes equations, we refer the Reader to [45]. For example, for the implementation in the Nektar1D solver [32] where the Forward Euler method was used, the real pole states were obtained as

41 $$\begin{aligned} x_i(t_k) = x_i(t_{k-1}) + \Delta t\cdot [a_{r_{i}}x_i(t_{k-1}) + q(t_{k-1})]. \end{aligned}$$

A similar relationship holds for the coupled states associated with complex pole pairs. The two sets of equations (39) and (40) provide the total pressure at the *k*-th time step according to the Forward Euler method

42 $$\begin{aligned} p(t_k) = p_r(t_k) + p_c(t_k) + c_0 q(t_k) \end{aligned}$$

in terms of flow rate at present and past time steps $q(t_k)$, $q(t_{k-1})$, and instrumental state variables $x_i(t_{k-1})$, which must be stored to enable the evaluation of the recurrence relations (41). We remark that the above implementation provides a direct extension of the actual implementation of 3WK boundary conditions in the solver Nektar1D [32].

Alternatively, since the proposed estimation method represents the model by means of a transfer function, the latter can be used directly into dedicated solvers for the simulation of dynamical systems, such as Simulink [46], which are also widely used in cardiovascular modeling.

## Results

This section provides numerical results for the experiments related to boundary conditions estimation based on Time-Domain Vector Fitting. The corresponding TDVF code template is available in [47]. In particular, after a general description in "Experimental Setup" section of the experimental setup, in "Estimation of Windkessel Boundary Conditions" section we evaluate the ability of the proposed method to estimate the parameters of 3WK models, compared to two other methods presented in the literature, and we assess the level of accuracy obtained when these models are used as boundary conditions in place of a more detailed vascular model. Then, we quantify the sensitivity of the obtained estimates to noise in "Sensitivity to Noise" section and their validity under changes of the physiological state of the patient ("Validity of BCs Estimated with Vector Fitting in Case of Varying Cardiac Output" section). Lastly, in "Higher-Order Boundary Conditions" section we evaluate the accuracy and robustness of the proposed algorithm for the estimation of higher order models.

### Experimental Setup

Experiments were conducted on a 1D arterial network representing the 55 largest arteries, as depicted in the left panel of Fig. 1. One-dimensional models provide an accurate approximation of blood flow in larger arteries, as documented in [48, 49], with a significant reduction in the computational cost with respect to 3D fluid–structure interaction (FSI) simulations. The parameters characterizing each segment are reported in [32], and refer to a normotensive case. The inlet boundary condition corresponds to a realistic inlet flow at the aortic root [32], while the outlet boundary conditions at each terminal vessel consist of a 3WK model, whose parameters are detailed in [32]. The blood flow in the 55-artery network was simulated using the Nektar1D solver [32], which solves the nonlinear, one-dimensional blood flow equations in a given network of compliant vessels. Specifically, Nektar1D adopts the method of characteristics and a discontinuous Galerkin numerical scheme [32] to solve numerically the system of equations. The coupling between 1D model segments and 0D models is obtained in Nektar1D by solving a Riemann problem at the 1D-0D interface [32, 50]. The solution provided by Nektar1D on the 55-artery network represents the reference solution for the model.

The 55-artery model was then reduced to a 21-artery model, containing only segments from the aorta up the first generation of bifurcations, by substituting the remaining segments with lumped parameter boundary conditions. A representation of the reduced model is shown on the right of Fig. 1, where the boundary conditions are represented as 3WK models. The original network on the left was truncated at the end of segments 3 (brachiocephalic artery), 15 (left common carotid artery), 19 (left subclavian artery), 29 (celiac artery), 42 (left common iliac artery), and 43 (right common iliac artery). The parameters of the corresponding lumped parameter terminations were estimated with the TDVF algorithm presented in "Methodology" section  through the following steps:

- the blood flow in the network described by the 55-artery model was simulated using Nektar1D, providing the reference solution of the model;
- the results of the simulation were used to extract the pressure and flow rate waveforms at the truncation sites;
- for each truncation location, pressure and flow rate data were fed into the TDVF algorithm, which estimated simultaneously the parameters of the lumped boundary conditions, as explained in "Methodology" section;
- the segments downstream to the truncation site were substituted with the estimated boundary conditions;
- the reduced 21-artery model obtained in this way was simulated using Nektar1D.

### Estimation of Windkessel Boundary Conditions

The results obtained from the estimation of Windkessel parameters with TDVF have been compared to those obtained with two other methods proposed in the literature. The first was presented in [21], and selects parameters of the 3WK models such that the net resistance and total compliance of the entire system are preserved. The method was reproduced by implementing the equations reported in [21]. The second one is based on the use of the *fminsearch* algorithm in MATLAB, which employs the Nelder-Mead simplex algorithm [33] to find the minimum of a given function. In particular, the minimization problem is defined as

43 $$\begin{aligned}{} & {} \min _{R_1,R_2,C,P_d} \Big \Vert p(t)- R_1q(t) + \frac{1}{C}\int _{0}^t e^{-\frac{1}{R_2C}(t-\tau )} q(\tau ) d \tau \nonumber \\{} & {} \quad + \frac{P_d}{R_2C}\int _{0}^t e^{-\frac{1}{R_2C}(t-\tau )}\theta (\tau ) d\tau \Big \Vert ^2 \end{aligned}$$

**Fig. 7:**
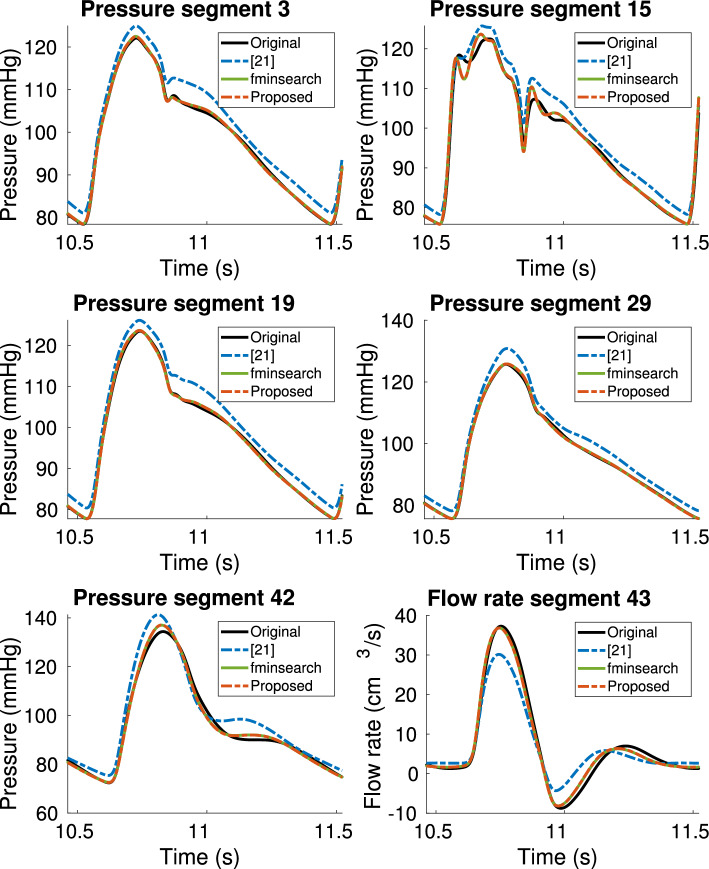
Comparison between the reference solution from the complete 55-artery model (solid black), reduction method from [21] (dashed blue), *fminsearch* method (solid green green), and proposed method (dashed red) for the three-element Windkessel boundary condition

Equation (43) can be derived by transforming (2) back into time domain, and expressing the input *q*(*t*) and the Heaviside function $\theta (t)$ by means of recursive convolutions. The four unknown parameters $R_1$, $R_2$, *C* and $P_d$, which were determined by means of *fminsearch*, were normalized to obtain a faster convergence of the algorithm. Figure 7 displays the obtained pressure waveforms at the truncation locations of the model, comparing the reference solution from the 55-artery model (black curve) to those from the reduced 21-artery model with 3WK parameters obtained with the technique presented in [21] (dashed blue curve), with *fminsearch* (solid green curve), and with the proposed method (dashed red curve). The curves obtained with *fminsearch* and the proposed method represent the best approximation of the original responses. The average and maximum errors for the pressure curves displayed in Fig. 7 are reported in Table 1: the results obtained with *fminsearch* and the proposed method are comparable in terms of accuracy, and with average errors always lower than 1.1%, up to one order of magnitude smaller than the alternative method proposed in [21]. The latter does not provide an estimation of $P_d$, so the original value of 10 mmHg used in the 55-artery model was maintained for all outlets. This choice causes a visible offset of the obtained pressure curves with respect to the original curves, noticeable in Fig. 7, confirming the necessity to estimate $P_d$ from measurements at each truncation point, instead of setting it to a fixed value common to all outlets. A comparison of the 3WK parameters obtained with the different methods at each truncated segment is reported in Table 2.

Even if the *fminsearch* method is a valid solution for estimating Windkessel parameters, its extension to higher order models is problematic, as nonlinear optimization methods become increasingly time-consuming and prone to the issue of local minima as order increases.

More importantly, the user would need to choose a representation of the model to define a suitable cost function that will be minimized, as in (43). Using the pole-residue representation, for example, would require to know the exact number of real and complex poles beforehand. It would be even more difficult to set a specific topology for the lumped circuit, just knowing the model response.

**Table 1:** Approximation errors for pressure curves at truncation locations, Windkessel case ("Estimation of Windkessel Boundary Conditions" section)

Segment	Method	Max error (%)	Avg error (%)
3	[21]	5.1	3.08
	*fminsearch*	0.67	0.30
	Proposed	0.67	0.30
15	[21]	6.51	3.24
	*fminsearch*	4.40	0.89
	Proposed	4.30	0.88
19	[21]	4.85	3.15
	*fminsearch*	0.63	0.30
	Proposed	0.62	0.30
29	[21]	4.31	3.12
	*fminsearch*	0.77	0.25
	Proposed	0.76	0.26
42–43	[21]	9.16	4.2
	*fminsearch*	2.71	1.1
	Proposed	2.81	1.1

**Table 2:** Comparison of estimated Windkessel parameters at the outlets of the 21-artery model ("Estimation of Windkessel Boundary Conditions" section)

Seg.	Method	$R_1$ (Pa s $\hbox {m}^{-3}$)	$R_2$ (Pa s $\hbox {m}^{-3}$)	*C* ($\hbox {m}^3$ $\hbox {Pa}^{-1}$)	$P_d$ (kPa)
3	[21]	0.18$\cdot$10$^8$	9.26$\cdot$10$^8$	9.70$\cdot$10$^{-10}$	1.33
	*fminsearch*	0.27$\cdot$10$^8$	8.46$\cdot$10$^8$	10.5$\cdot$10$^{-10}$	1.54
	Proposed	0.26$\cdot$10$^8$	8.43$\cdot$10$^8$	10.5$\cdot$10$^{-10}$	1.58
15	[21]	3.60$\cdot$10$^8$	19.2$\cdot$10$^8$	1.14$\cdot$10$^{-10}$	1.33
	*fminsearch*	6.72$\cdot$10$^8$	14.2$\cdot$10$^8$	1.31$\cdot$10$^{-10}$	1.58
	Proposed	6.55$\cdot$10$^8$	14.2$\cdot$10$^8$	1.23$\cdot$10$^{-10}$	1.64
19	[21]	1.00$\cdot$10$^8$	17.0$\cdot$10$^8$	5.39$\cdot$10$^{-10}$	1.33
	*fminsearch*	0.67$\cdot$10$^8$	15.5$\cdot$10$^8$	6.17$\cdot$10$^{-10}$	1.59
	Proposed	0.67$\cdot$10$^8$	15.4$\cdot$10$^8$	6.13$\cdot$10$^{-10}$	1.68
29	[21]	1.62$\cdot$10$^8$	7.58$\cdot$10$^8$	3.06$\cdot$10$^{-10}$	1.33
	*fminsearch*	1.99$\cdot$10$^8$	6.90$\cdot$10$^8$	4.36$\cdot$10$^{-10}$	1.41
	Proposed	1.99$\cdot$10$^8$	6.91$\cdot$10$^8$	4.36$\cdot$10$^{-10}$	1.41
42-43	[21]	1.57$\cdot$10$^8$	14.8$\cdot$10$^8$	5.04$\cdot$10$^{-10}$	1.33
	*fminsearch*	0.98$\cdot$10$^8$	13.6$\cdot$10$^8$	6.11$\cdot$10$^{-10}$	1.61
	Proposed	0.97$\cdot$10$^8$	13.5$\cdot$10$^8$	6.06$\cdot$10$^{-10}$	1.71

### Sensitivity to Noise

**Fig. 8:**
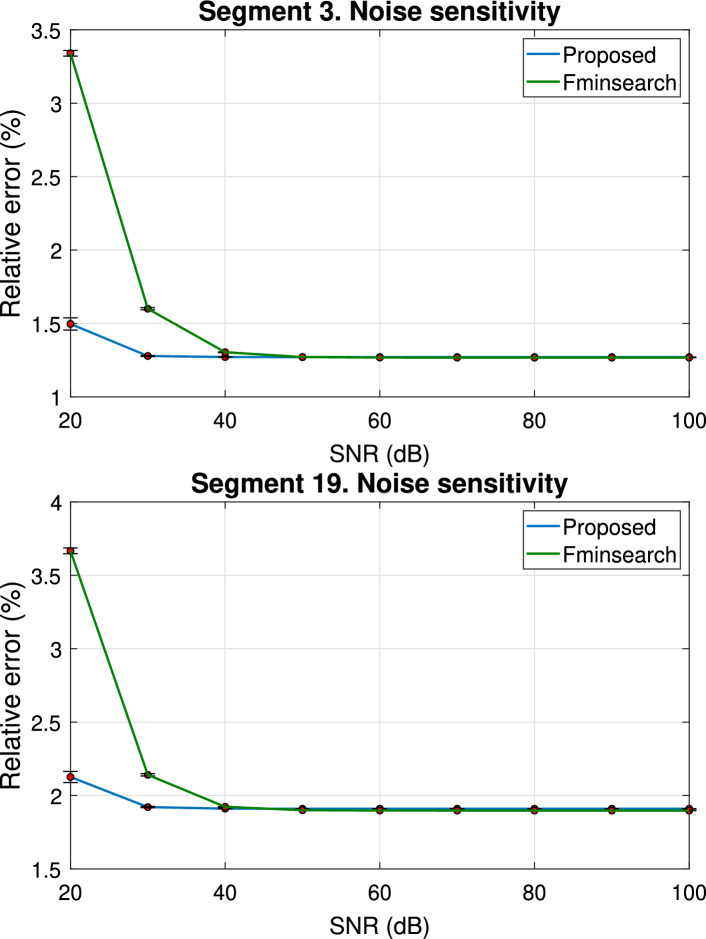
Relative error between pressure samples obtained from the reference solution of the 55-artery network and Windkessel models estimated from noisy data, with different SNR values. The Windkessel parameters were estimated with TDVF (blue curve) and *fminsearch* (green curve). Vertical bars in correspondence of the different SNR values indicate the standard deviation of the relative error

To investigate the robustness of the proposed algorithm, both pressure and flow rate data were corrupted with zero-mean white Gaussian noise with signal-to-noise ratio (SNR) ranging from 20 dB up to 100 dB, corresponding to a noise standard deviation ranging between 3.95 mmHg and 3.90$\cdot 10^{-4}$ mmHg for pressure and 1.18 $\hbox {cm}^3$/s and 1.15$\cdot 10^{-4}$ $\hbox {cm}^3$/s for flow rate, respectively. For each SNR level, we generated 50 different noise realizations to corrupt the data. Then, for each corrupted dataset a 3WK boundary condition was estimated, both with the proposed method and *fminsearch*. The results for this analysis are reported in Fig. 8, where the relative error between the pressure samples from the 55-artery network and the output of the Windkessel models estimated at different SNR values is reported. In this experiments, the relative error is computed as

44 $$\begin{aligned} \frac{\Vert p(t)-p_M(t)\Vert _2}{\Vert p(t)\Vert _2}, \end{aligned}$$

where *p*(*t*) is the reference noiseless pressure signal and $p_M(t)$ is the corresponding model reconstruction.

**Fig. 9:**
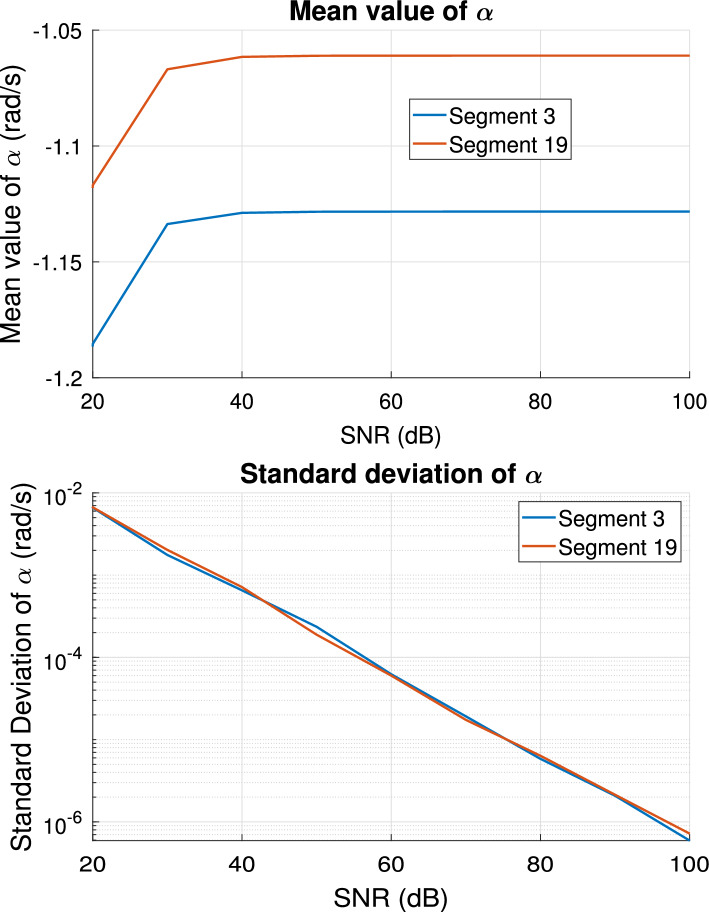
The mean value (top panel) and the standard deviation (bottom panel) of the pole $\alpha$ estimated by TDVF algorithm as a function of the SNR level

Moreover, at each SNR value, the bar indicates the standard deviation. Both techniques are able to estimate the correct boundary conditions starting from data samples with SNR ranging from 100 dB down to 40 dB, without any loss of accuracy. For segment 19, the error at 20 dB and 30 dB levels is about $2.1\%$ and $1.9\%$ respectively, while for segment 3 these errors are $1.5\%$ and $1.3\%$, with the proposed approach performing slightly better that *fminsearch*. Additionally, we report in Fig. 9 the mean value and the standard deviation of the pole $\alpha$ estimated by the TDVF algorithm, for each considered level of SNR, computed over models obtained for the 50 different noise realizations. These results verify the numerical robustness of the proposed estimation also in presence of noisy data, a condition more likely to occur when using patient-specific measurements instead of simulation results to drive the boundary conditions estimation. Patient-specific measurements, however, may vary within an individual due to various physiological factors, which are not considered in this sensitivity analysis.

### Validity of BCs Estimated with Vector Fitting in Case of Varying Cardiac Output

**Fig. 10:**
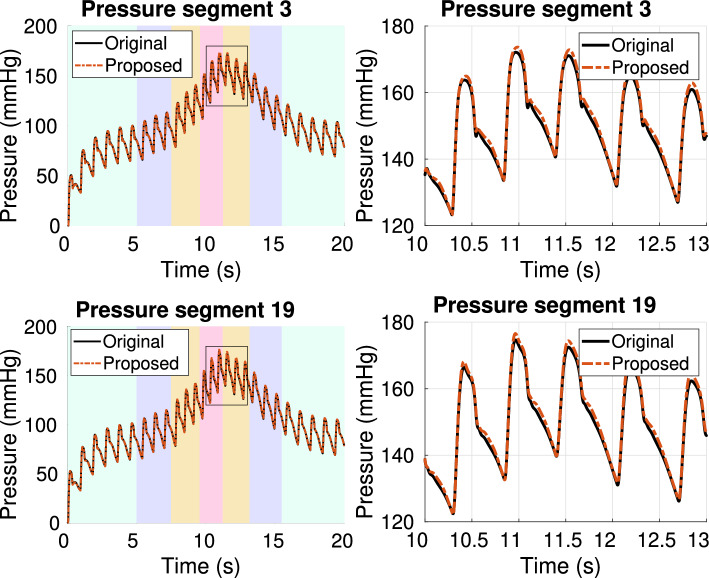
Pressure waveforms at segment 3 (top) and 19 (bottom) for the case of varying cardiac output. Background color indicates the corresponding level of stress: relaxation (light blue), baseline (purple), medium stress (orange), high stress (pink). The plots on the right zoom on the black rectangle displayed on the plots on the left. Black lines refer to the reference 55-artery model, while red dashed lines refer to the reduced 21-artery model

In the previous section, boundary conditions were estimated from data coming from a simulation of the cardiovascular system under normal conditions. However, under certain circumstances like physical exercise, the cardiovascular system does not operate under normal conditions anymore, experiencing physiological changes in heart rate and cardiac output. These conditions can be modeled by properly changing the flow rate at the aortic root, which is the input imposed on the 55-artery model used in this work. Therefore, it is important to verify that the boundary conditions estimated with the standard input flow rate are still valid in presence of physiological changes of the cardiac output. In order to do so, we emulated a realistic variation of the input aortic flow rate under mental stress conditions by using the dataset presented in [34], which provides different aortic root flow rates corresponding to different levels of mental stress in a human subject. These conditions translate into increased peak velocity and acceleration due to the increase in ejection fraction during stress [35]. An input flow corresponding to different levels of mental stress was then generated and used as input for both the 55-artery model, chosen as a reference, and the 21-artery one. In the latter, the 3WK BCs previously estimated with the proposed approach in the normotensive case, and reported in Table 2, were used. Pressure waveforms at different points of the model are reported in Fig. 10, where the results in the reference 55-artery model (black line) are compared to those in the 21-artery model. The background colors in the left panel of Fig. 10 indicate the corresponding level of mental stress induced by the input aortic flow rate, varying from a relaxed state (light blue), to the baseline (purple), medium (orange) and high (pink) levels of mental stress. The corresponding heart rate (HR) and cardiac output (CO) associated to each stress level are reported in Table 3 and can be found in [34]. From Fig. 10 it is possible to see that the reduced model is able to closely follow the changes caused by the varying input flow, with average relative errors smaller than 0.7% for both segments. The results confirm that the estimated boundary conditions are valid also when considering a transient situation, instead of a periodic one.

**Table 3:** Prescribed heart rate (HR) and Cardiac output (CO) for the four different levels of stress, as described in "Validity of BCs estimated with Vector Fitting in case of varying cardiac output" section

Stress level	HR (bpm)	CO (l $\hbox {min}^{-1}$)
Relaxation	66.9	5.2
Baseline	74.9	6.2
Medium stress	91.9	8.7
High stress	108.7	11.7

### Higher-Order Boundary Conditions

**Fig. 11:**
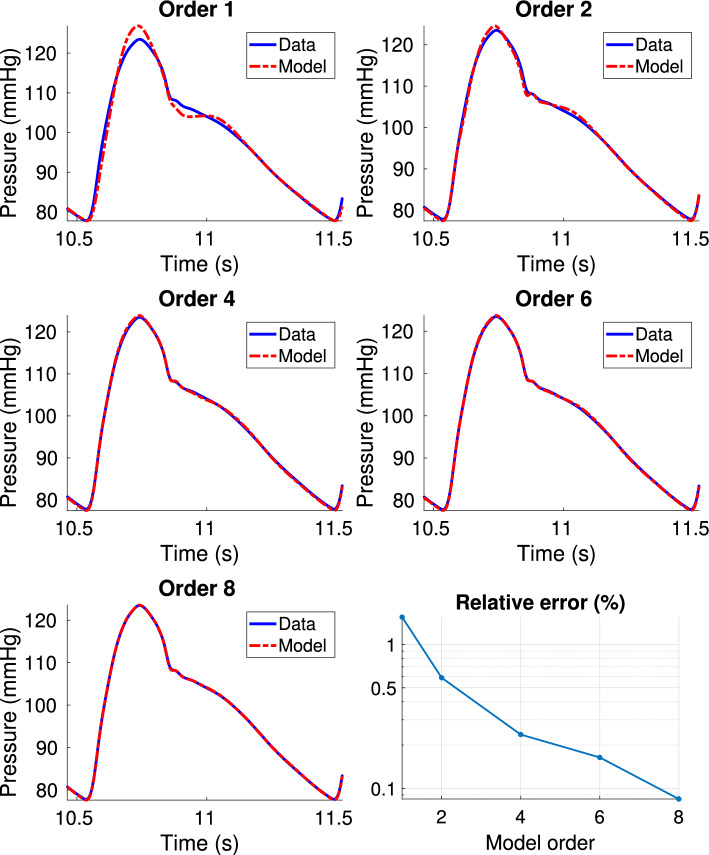
Comparison of pressure waveform in segment 19 (blue curve) against models with different order obtained with Vector Fitting. The bottom-right panel reports the relative error (44) on pressure vs model order, computed over one period at steady-state

**Fig. 12:**
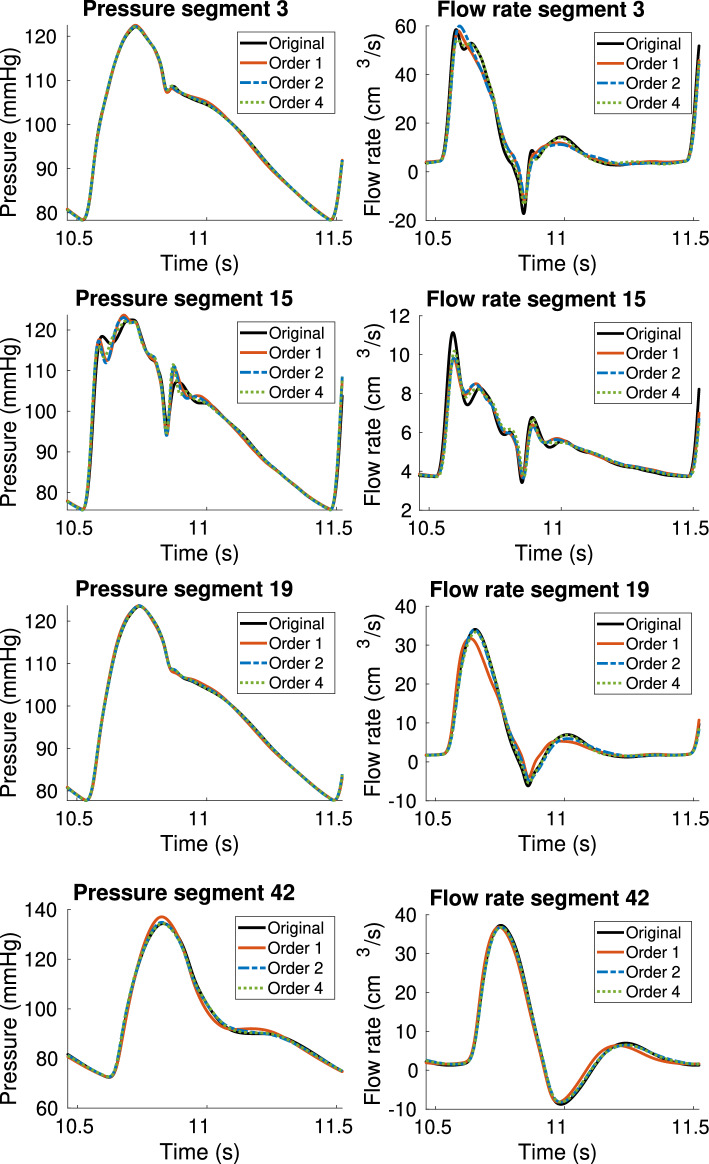
Comparison of pressure and flow waveforms in the 55-artery model (solid black curve) and in the 21-artery one with boundary conditions of different orders, estimated with Vector Fitting

In this section, we test the use of the proposed technique for the estimation of higher order boundary conditions and we investigate their accuracy compared to standard 3WK models. The same experimental setup presented in 3.1, consisting of the reference 55-artery model and the reduced 21-artery model, was adopted. In Table 4, we report the number of iterations needed by TDVF to attain convergence. In Fig. 11, we first compare the reference pressure from the 55-artery model used for the estimation (blue line), to the pressure estimated by the proposed model (dashed red line), for the same flow rate coming from the 55-artery model. The results reported in Fig. 11 refer to the pressure in segment 19 fitted with models of order up to 8 (comparable results were obtained for the other segments). These can be defined as “a-priori” results of the TDVF models alone, which aim to assess the quality of fit for different model orders, before using them as boundary conditions of the circulation network. It is clear from Fig. 11 that accuracy greatly improves by increasing the model order. The right panel on the third line of Fig. 11 shows the average relative error on pressure versus model order, suggesting a decrease of around one order of magnitude going from order 1 to order 8. We then used the estimated models as boundary conditions for the reduced 21-artery model, simulated with Nektar1D. This step required a modification of the solver to accept boundary conditions defined as in 2.2.3, that we performed as discussed in "Implementation of High-Order Boundary Conditions" section. Pressure and flow rate curves up to order 4 at the truncated segments of the reduced 21-artery model are displayed in Fig. 12, while the relative errors on pressure and flow rate waveforms up to order 8 are reported in Tables 5 and 7 (average error), and in Tables 6 and 8 (maximum error). It is clear, both from plots and from numerical results, that higher order boundary conditions can model pressure and flow rate more accurately than a simple Windkessel (corresponding to order 1). In particular, a significant improvement can be seen with order 2 and order 4, where average errors can decrease up to one order of magnitude with respect to BCs of order 1. Orders above 4, instead, did not seem to provide an improvement in terms of accuracy. Segment 15 is the only case which does not seem to benefit from higher order boundary conditions, with the error remaining nearly constant for both pressure and flow rate, even for higher orders. This is further confirmed by the lack of stabilization of the pole relocation iteration, which is stopped not upon convergence but upon hitting the maximum allowed number of iterations $\nu _{\max }=100$, see Table 4. Looking at the corresponding plots in Fig. 12, it can be noticed that the curves obtained after the truncation are qualitatively different from the original pressure and flow in 55-artery model (black curve). A possible cause could be the higher wall viscosity of segment 15 with respect to the other terminal segments, which could increase the presence of nonlinear effects, hard to model with a linear boundary condition. However, no conclusive explanation was reached.

As already mentioned in "Methodology" section, the adoption of higher order models requires only a negligible increase in the computational cost. Table 9 reports the wall clock time for the Vector Fitting step and for the 21-segments model simulation with Nektar1D, for different model orders. In the first column (Single TDVF), the average time and standard deviation for the estimation of a single boundary condition is reported, which was obtained by averaging over the estimation times at the six truncation locations of the 55-artery model. The second column (Total TDVF) reports the total time required to estimate the six boundary conditions necessary to move from the 55-artery model to the 21-artery one. The last column reports the time required by Nektar1D for the simulation of the 21-artery model. All the simulations were run on a laptop with Intel Core i7-7700HQ CPU, 2.80 GHz, and 16 GB RAM. As verified in [51], the computational cost of the Vector Fitting algorithm scales linearly with the number of poles. It is worth noticing how the total time required by TDVF for the estimation is less than that required for the simulation by at least one order of magnitude, thus constituting a very small computational overhead. Moreover, the Nektar1D simulation time does not increase when increasing the model order, confirming that the use of higher order boundary conditions comes at no additional computational cost.

**Table 4:** Number of iterations performed by TDVF to attain convergence for the higher order boundary conditions models.("Higher-Order Boundary Conditions" section)

Segment	Number of iterations				
	Order 1	Order 2	Order 4	Order 6	Order 8
3	4	79	33	36	49
15	9	100	100	100	100
19	6	23	65	39	19
29	6	13	44	54	39
42-43	6	18	167	27	28

**Table 5:** Average relative errors (%) on pressure at truncation locations, with models of higher order ("Higher-Order Boundary Conditions" section)

Segment	Average relative error (%)				
	Order 1	Order 2	Order 4	Order 6	Order 8
3	0.30	0.12	0.078	0.084	0.079
15	0.88	0.75	0.65	0.67	0.67
19	0.30	0.1	0.069	0.079	0.068
29	0.26	0.15	0.11	0.093	0.079
42-43	1.1	0.36	0.31	0.23	0.21

**Table 6:** Maximum relative errors (%) on pressure at truncation locations, with models of higher order ("Higher-Order Boundary Conditions" section)

Segment	Maximum relative error (%)				
	Order 1	Order 2	Order 4	Order 6	Order 8
3	0.67	0.59	0.46	0.53	0.51
15	4.30	4.79	5.16	5.01	4.80
19	0.62	0.44	0.33	0.34	0.35
29	0.76	0.61	0.44	0.41	0.42
42-43	2.81	1.33	1.17	1.07	0.94

**Table 7:** Average relative errors (%) on flow rate at truncation locations, with models of higher order ("Higher-Order Boundary Conditions" section)

Segment	Average relative error (%)				
	Order 1	Order 2	Order 4	Order 6	Order 8
3	3.14	3.63	1.80	1.70	1.60
15	1.90	2.1	2.0	1.94	1.90
19	3.41	1.67	1.0	0.95	0.84
29	0.8	0.37	0.21	0.19	0.17
42-43	2.5	1.07	0.97	0.61	0.58

**Table 8:** Maximum relative errors (%) on flow rate at truncation locations, with models of higher order ("Higher-Order Boundary Conditions" section)

Segment	Maximum relative error (%)				
	Order 1	Order 2	Order 4	Order 6	Order 8
3	12.05	14.67	10.96	11.83	10.80
15	13.71	15.61	16.47	16.12	15.61
19	15.03	6.53	4.29	3.46	3.58
29	4.45	1.22	0.92	0.98	0.88
42-43	6.06	2.22	1.89	1.54	1.40

**Table 9:** Measured wall clock times for the estimation of a single boundary condition with Vector Fitting (Single TDVF), for the estimation of the boundary conditions at the six truncation sites of the 55-artery model (Total TDVF), and for the simulation of the reduced 21-segments model with Nektar1D. Results are reported for different model orders

Model order	Single TDVF (s)		Total TDVF (s)	Nektar1D (s)
	Mean	Std dev		
1	1.25	0.15	7.52	471
2	1.42	0.20	8.53	473
4	1.98	0.25	11.86	470
6	1.99	0.38	11.97	470
8	2.54	0.64	15.23	472

## Discussion

Results presented in "Results" section show that Time-Domain Vector Fitting is able to estimate accurate lumped boundary conditions, making it a promising tool for cardiovascular modeling. When employed to estimate Windkessel boundary conditions, Vector Fitting was able to accurately determine optimal values for the Windkessel parameters. The efficacy of the proposed approach was further assessed in presence of noisy synthetic measurements, where TDVF provided accurate parameters starting from data with up to 20 dB of SNR, and under physiological changes of pressure and flow rates induced by changing levels of mental stress. The results obtained with Vector Fitting were comparable to those attained with two other estimation methods presented in the literature. The advantage of the proposed approach, however, is mainly its ability to estimate an increasing number of parameters simultaneously and automatically. The proposed model parametrization based on the use of transfer functions, in fact, can be used to describe any linear dynamical system, allowing to generalize the model to differential relations of arbitrary order. The alternative solutions for higher order BCs proposed in the literature, instead, resort to specific circuit topologies, from which a generalization is difficult to obtain. Thanks to the aforementioned properties of the TDVF method, it was possible to formulate and estimate in a systematic way boundary conditions of increasing order with limited additional computational cost. For the case under analysis, consisting of a 55-artery model reduced to 21 arterial segments, boundary conditions with order up to 8 were estimated and compared, in order to assess the effect that the order of the boundary condition has on its ability to accurately approximate the downstream vasculature. Even if in the scenarios presented in the paper the estimated boundary conditions have all the same order, this is not a requirement for the method. In fact, as each boundary condition is estimated with TDVF independently, different boundary conditions can be used in different points of the model. Results showed that an order of 2 provides a significant increase in accuracy with respect to BCs of order 1, the most common choice up to now in the form of Windkessel models. Orders above 4, instead, provided negligible improvements in terms of accuracy in the model of the systemic arterial system considered.

### Limitations and Future Developments

The estimation of boundary conditions with the proposed method requires time samples of both pressure and flow rate at the truncation location. This requirement could be a limitation, depending on whether pressure and flow rate measurements are available at the same location or not. The following scenarios may arise:

1. flow rate and pressure are available at the same location. This is typically the case when the BC is meant to be derived from a circulatory model, such as the 1D models used in this work. It is also the case where an in-vivo pressure measurement is performed with a catheter, on purpose or as part of the clinical procedure;
2. flow rate is available, but pressure information is unavailable, or available only at a different location (e.g. in the arm). This scenarios is common in clinical practice where phase-contrast or 4D-flow MRI can measure the flow rate at the truncation location, but only cuff pressure is acquired. In this case, the pressure at the truncation location can be estimated from: (i) brachial diastolic and systolic pressure, as commonly done to estimate Windkessel model parameters; (ii) using pressure waveform generators [12, 13]; (iii) from blood velocity using recent advancements in 4D-flow MRI processing [15–17]. We are also investigating how to generalize the proposed method to estimate the BC given pressure information at an alternative location, and we believe that under mild assumptions (e.g. linearity) this should be feasible [52–54];
3. neither flow rate nor pressure measurements are available. In this case, both quantities have to be estimated using literature information or waveform generators. The main issue in this scenario is the limited information available, not how to determine BC coefficients. In this scenario, a low-order BC (e.g. a 2- or 3-element Windkessel model) is typically advisable, and the proposed method can be used as an automated and robust way to determine the Windkessel model coefficients.

The use of the proposed automated approach for the estimation of boundary conditions removes the uncertainty associated with empirical methods relying on manual tuning and a trial and error approach. The proposed method, in due course, can also guarantee remarkable robustness to the the limited accuracy of clinical measurements, that are inevitably affected by significant measurement errors. It should be noted that there are still other sources of uncertainty, such as those arising from an imperfect reconstruction of the anatomy from medical images, from errors in the measurement and reconstruction of the pressure and flow rates, and from the limited understanding of self-regulation mechanisms present in the system circulation.

When the proposed approach is applied to estimate low-order boundary conditions, as in "Estimation of Windkessel Boundary Conditions" section, the result is a standard Windkessel model that retains its standard physical interpretation and parameterization. When a high-order BC is required, the proposed method provides a suitable differential relation between pressure and flow-rate that describes the hemodynamic behaviour of the region downstream the outlet. If a physical interpretation is required, the proposed model (18) can be represented as an equivalent electrical circuit using network synthesis algorithms [9]. This conversion provides some physical insight into the obtained BC, and a potential opportunity for parametrizing the BC coefficients based on clinical information, such as patient age, or presence of hypertension. As an alternative approach to parametrization, the proposed method can be potentially generalized to make the proposed BCs parametrized by other clinical parameters, as successfully done for vector fitting type algorithms in other application areas [55].

Finally, we remark that, in this work, the vector fitting method has been tested only on 1D models of the cardiovascular system. The extensions to three-dimensional models will be the subject of future developments.

## Conclusions

In this work, we proposed a new automated method based on the Time-Domain Vector Fitting algorithm for the estimation of boundary conditions for cardiovascular models. Starting from pressure and flow rate samples at the truncation location, this method can estimate boundary conditions corresponding to differential equations of increasing order. First, the TDVF algorithm was used to automatically estimate 3WK boundary conditions, starting from a 1D model comprising the 55 main arteries of the human arterial system. The robustness of the estimation procedure was verified in presence of noisy data, with down to 20 dB of signal-to-noise ratio, and in presence of physiological changes of pressure and flow rate induced by high levels of mental stress. Second, we proposed a generalization of the three-element Windkessel model to obtain boundary conditions of arbitrary order. We estimated higher order boundary conditions with TDVF, and we investigated the improvement in accuracy they provide with respect to the 3WK model. On the 55-artery model, experimental results showed that boundary conditions up to order 4 are able to model the downstream pressure and flow rate more accurately than the Windkessel model, while orders above 4 provided negligible improvements in term of accuracy. Future works will aim at applying the proposed methodology to generate boundary conditions for three-dimensional CFD simulations, as well as to use TDVF to generate higher order boundary conditions even in absence of co-located measurements of pressure and flow rate.
